# Pneumomediastinum, Pneumoretroperitoneum, Pneumoperitoneum and Subcutaneous Emphysema Secondary to a Penetrating Anal Injury

**DOI:** 10.3390/diagnostics11040707

**Published:** 2021-04-15

**Authors:** Yu-Ting Hsiao, Shyh-Wen Lin, Pei Wen Chuang, Ming-Jen Tsai

**Affiliations:** Department of Emergency Medicine, Ditmanson Medical Foundation Chiayi Christian Hospital, Chiayi 600, Taiwan; amy810129@gmail.com (Y.-T.H.); linsw0459@yahoo.com.tw (S.-W.L.); sky.tomoato@msn.hinet.net (P.W.C.)

**Keywords:** pneumoperitoneum, pneumoretroperitoneum, pneumomediastinum, subcutaneous emphysema

## Abstract

Simultaneous occurrence of pneumoperitoneum, pneumoretroperitoneum, pneumomediastinum and subcutaneous emphysema is rare. The most reported mechanisms are barotrauma, thermal injury and instrument puncture caused by colonoscopy. Ectopic air may travel into different body compartments through distinct anatomical fascial planes. Definite curative treatment involves surgical repair of the bowel wall defect. Conservative treatment is available in selected patients. Here, we present a case of traumatic penetrating rectal injury leading to developing air in the peritoneum, retroperitoneum, mediastinum, and subcutaneous space with good recovery under conservative treatment.

A 62-year-old man presented with anal pain and chest discomfort for one day stated that he had a penetrating anal injury after a fall while working in the field. He removed the foreign body by himself. Due to persistent pain and lethargy, he was brought to the emergency department by his family. Physical examination showed a wound of the perianal area (Figure 1) and palpable crepitus in the left lower chest wall. The abdomen was soft without tenderness. He had smooth respiration and his vital signs were normal. Abnormal laboratory findings included leukocytosis (14,050/µL) and marked elevated C-reactive protein levels (15.9 mg/dL). Chest and abdominal X-rays were performed (Figure 2). Computed tomography (CT) was arranged subsequently (Figure 3 and Figure 4). Pneumoperitoneum, pneumoretroperitoneum, pneumomediastinum and subcutaneous emphysema were observed on the chest and abdomen CT. The radiologist suggested the diffused air distribution among different body parts originated from traumatic rectal perforation. Treatment was initiated with fluid and third-generation cephalosporin. A specialist was consulted for surgical intervention. However, the patient refused surgery and hospitalization. He was discharged against medical advice on the second day. Oral moxiflocaxin was prescribed and an outpatient appointment was arranged. On the fifth day, he returned to the outpatient department and was hospitalized due to persistent anal pain. After hospitalization, he received systemic antibiotics with ertapenem, fasting, and intravenous nutrition. He started eating on the eighth day. On the 11th day, only some residual peritoneal air was found following CT scan. The patient was uneventful and was discharged after 6 days of hospitalization.

Simultaneous occurrence of pneumoperitoneum, pneumoretroperitoneum, pneumomediastinum and subcutaneous emphysema is rare. The most commonly reported mechanisms are barotrauma, thermal injury and instrument puncture caused by colonoscopy [1,2,3]. Depending on the site of perforation, intraluminal air may escape into the peritoneal or retroperitoneal space [1,2]. The ectopic air may travel into different body compartments through distinct anatomical fascial planes [1]. Retroperitoneal air may pass into the mediastinum through esophageal or aortic hiatus of the diaphragm [1,2,3,4]. Subcutaneous emphysema may occur when air travels along the mesentery to abdominal wall and then spreads to the chest wall [1]. In rare cases, pneumothorax can occur if the mediastinal parietal pleural ruptures [2]. There is no strong suggestion of particular treatment and the choice is based on a case-by-case basis [5,6]. Conservative treatment is acceptable in patients in good condition, with stable hemodynamics and no signs of peritonitis [5,6]. A literature review including 32 cases of extraperitoneal colonic perforation following colonoscopy reported that the most common site of perforation was the rectosigmoid colon and pneumomediastinum was the most common imaging finding [6]. Conservative treatment was successful in most patients (53%) in this review [6]. However, surgical intervention is indicated if there is evidence of peritonitis, fecal content leakage, and no improvement or worsening after conservative treatment [5,6]. Surgical options include repair of the bowel defect or segmental resection under laparotomy or laparoscopy [2,5,6]. If perforation cannot be located, an ileostomy can be created for decompression and help healing [2,5].

In our case, the patient suffered from penetrating traumatic rectal perforation developing pneumoperitoneum, pneumoretroperitoneum, pneumomediastinum and subcutaneous emphysema. Although laboratory abnormality raised the concern of ongoing sepsis, there was no evidence of peritonitis or hemodynamic instability. This patient had a good prognosis under conservative treatment. Physicians should be aware of patients with sudden abdominal pain, dyspnea, or chest pain following rectal injury or colonoscopy, because these might be the initial signs of rectal or colonic perforation [3]. In addition, the presence of ectopic air, such as pneumomediastinum, pneumoretroperitoneum, and subcutaneous emphysema comprise the initial imaging findings of extraperitoneal perforation of the colon or rectum. A high index of suspicion and a tailored treatment strategy are mandatory to manage this condition.

## Figures and Tables

**Figure 1:**
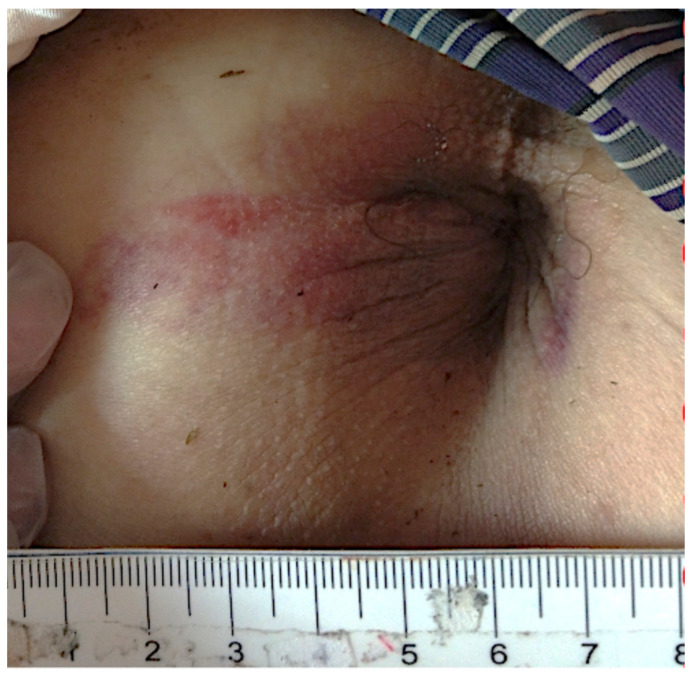
One wound was found at lateral perianal area of the patient.

**Figure 2:**
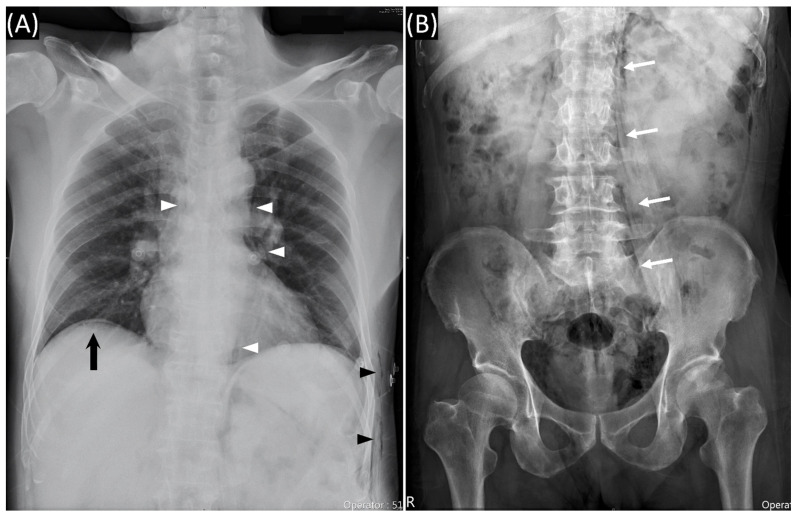
(**A**) Chest radiography showed subcutaneous emphysema (black arrowheads), pneumomediastinum (white arrowheads), and subdiaphragmatic free air (black arrow). (**B**) Abdominal radiography showed paraspinal air which outlines the psoas muscle (white arrows).

**Figure 3:**
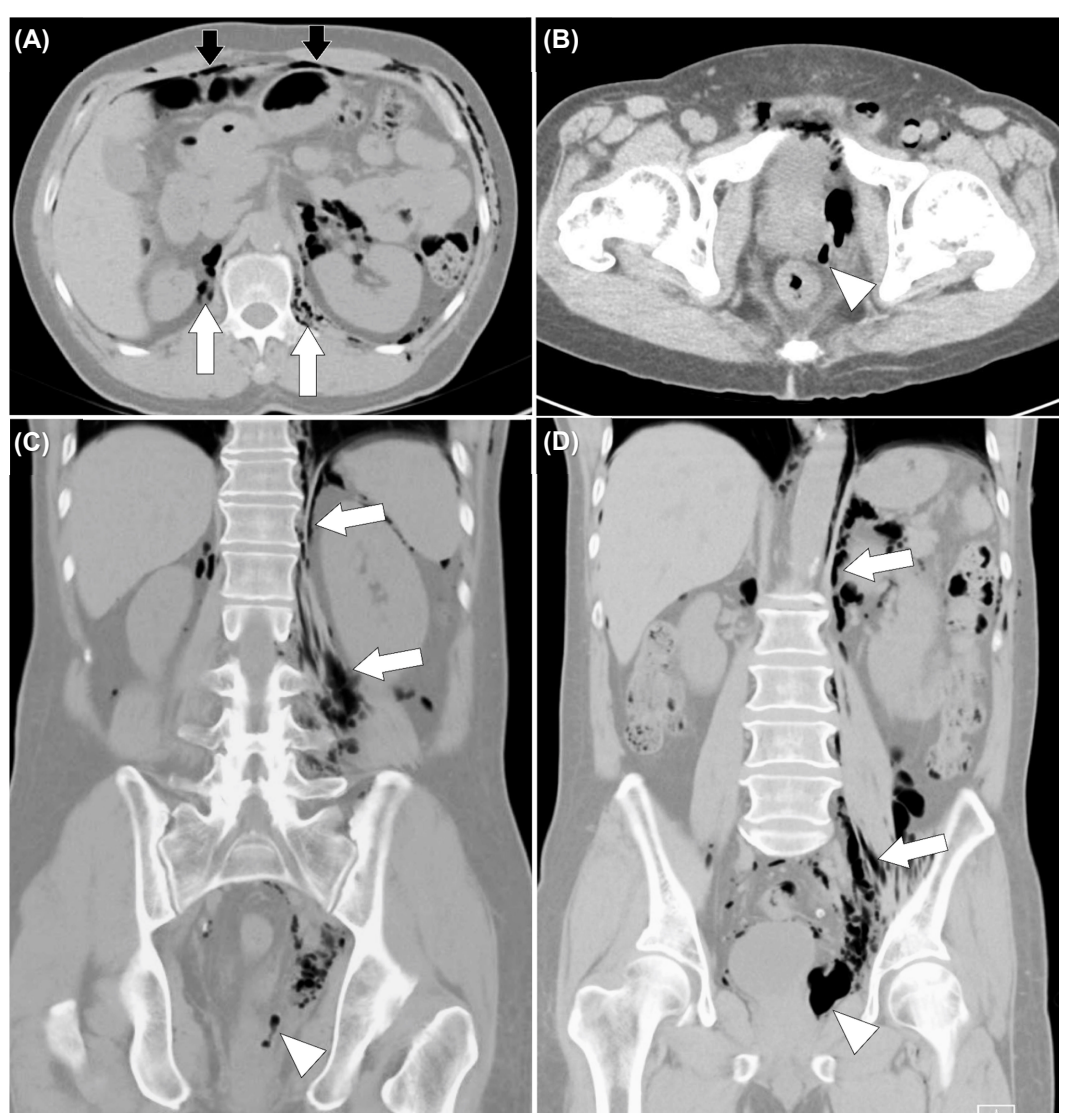
Non-enhanced computed tomography of the abdomen showed air tract extended from left peri-rectal area (white arrowheads) (**B**–**D**) to retroperitoneum (white arrows) (**A**,**C**,**D**), and peritoneum (black arrows) (**A**).

**Figure 4:**
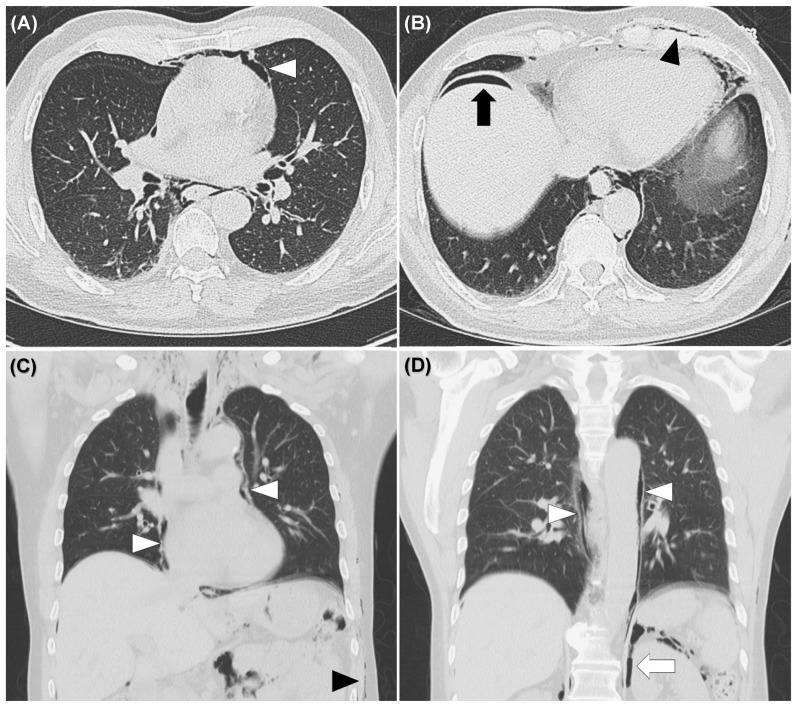
Non-enhanced computed tomography of the chest showed pneumomediastinum (white arrowheads) (**A**,**C**,**D**), subcutaneous emphysema (black arrowheads) (**B**,**C**), pneumoperitoneum (black arrow) (**B**), pneumoretroperitoneum (white arrows) (**D**), but no evidence of rib fracture and pneumothorax.

## Data Availability

The data that support the findings of this paper are available from the corresponding author, M.-J.T., upon reasonable request.
